# Remarkable Geographic Structuring of Rheophilic Fishes of the Lower Araguaia River

**DOI:** 10.3389/fgene.2018.00295

**Published:** 2018-08-14

**Authors:** Tomas Hrbek, Natasha V. Meliciano, Jansen Zuanon, Izeni P. Farias

**Affiliations:** ^1^Laboratório de Evolução e Genética Animal, Departmento de Genética, Instituto de Ciências Biológicas, Universidade Federal do Amazonas, Manaus, Brazil; ^2^Instituto de Saúde e Biotecnologia, Universidade Federal do Amazonas, Coari, Brazil; ^3^Coordenação de Biodiversidade, Instituto Nacional de Pesquisas da Amazônia, Manaus, Brazil

**Keywords:** Cichlidae, Anostomidae, Tocantins river basin, mitochondrial control region, micro endemism, diversity loss, rapids

## Abstract

Rapids and waterfalls, and their associated fauna and flora are in peril. With the construction of each new hydroelectric dam, more rapids and waterfalls are destroyed, leading to the disappearance of associated fauna and flora. Areas of rapids harbor distinct, highly endemic rheophilic fauna and flora adapted to an extreme environment. Rheophilic habitats also have disjunct distribution both within and across rivers. Rheophilic habitats thus represent islands of suitable habitat separated by stretches of unsuitable habitat. In this study, we investigated to what extent, if any, species of cichlid and anostomid fishes associated with rheophilic habitats were structured among the rapids of Araguaia River in the Brazilian Amazon. We tested both for population structuring as well as non-random distribution of lineages among rapids. Eight of the nine species had multiple lineages, five of these nine species were structured, and three of the eight species with multiple lineages showed non-random distribution of lineages among rapids. These results demonstrate that in addition to high levels of endemicism of rheophilic fishes, different rapids even within the same river are occupied by different lineages. Rheophilic species and communities occupying different rapids are, therefore, not interchangeable, and this realization must be taken into account when proposing mitigatory/compensatory measures in hydroelectric projects, and in conservation planning.

## Introduction

Brazil, as many other South American nations, has experienced a period of rapid economic growth accompanied by expanding energy needs. To meet its energetic needs, Brazil has invested heavily in hydroelectric power generation such that ≈ 80% of Brazil's electricity production is currently met by hydropower (International Energy Agency, 2013a). At the same time, it is estimated that up to 55% the hydroelectric potential still could be exploited (International Energy Agency, 2013b). While the hydroelectric potential of the Paraná and São Francisco river basins in southern, central and northeastern Brazil has largely been realized, the Amazon basin is the next and last hydroelectric frontier (Lees et al., 2016; Latrubesse et al., 2017). However, several large dams, such as the Tucuruí, Balbina, Santo Antônio, Jirau and the recently completed Belo Monte have already been implemented in the Brazilian Amazon. An additional 200+ dams, such as the Tapajós hydroelectric complex, have been proposed by South American governments (Finer and Jenkins, 2012; Castello et al., 2013; Lees et al., 2016). The construction of the Tapajós hydroelectric complex was only not initiated mostly due to the economic crisis Brazil is experiencing since 2014 (Fearnside, 2015). If these plans were to be enacted, only three Amazon tributaries would remain unimpounded (Castello and Macedo, 2016). Although hydroelectric projects have been lauded as cheap and clean energy alternatives, dam construction and operation result in substantial environmental stresses including the destruction of rheophilic habitats by permanently submerging rapids and waterfalls (Clausen and York, 2008; Castello and Macedo, 2016; Pelicice et al., 2017).

Rheophilic habitats are a unique geomorphological feature of Amazonian River tributaries that descend the Brazilian and Guiana shields, cutting through the rocky surface to create a series of waterfalls and rapids, and a complex matrix of rocky habitats. Rapids are characterized as river sections of supercritical flow, where surface tension breaks at the water/air interface (Hawkins et al., 1993); the presence of rocky substratum is a fundamental component of this type of environment. These river sections are characterized by not only high velocity but also by highly heterogeneous water flow, oxygen–rich waters, and a very complex substrate matrix consisting of rocky slabs, caves, cracks, and crevices with lodged tree trunks harboring a highly diverse array of niches. It is in these high–energy sections of the rivers where hydroelectric projects are developed. Damming of the river at these sites to create a reservoir (most Amazonian hydroelectric projects) or diverting the river from these sites into a holding reservoir (as done in the Belo Monte hydroelectric project), changes permanently the hydrology of the river. Rheophilic habitats generally are permanently submerged underneath the reservoir, turning a high–energy, oxygen–rich lotic habitat into an hypoxic lentic habitat. In the case of the Belo Monte hydroelectric project, much of the rheophilic habitat in the Volta Grande has become permanently dewatered (emerged), and those sections that had not, no longer are subject to seasonal water level fluctuations necessary for the flowering and thus the reproduction of the unique Podostemaceae flora and the fish fauna that depend on this resource and which use seasonal water level fluctuations as reproductive clues (Lowe-McConnell, 1987). Under both scenarios, the specialized rheophilic fauna and flora suffers local or potentially even global extinctions (Winemiller et al., 2016).

From an ichthiofaunistic perspective, rapids had historically been poorly sampled and are poorly known environments (Böhlke et al., 1978; Menezes, 1996). On the other hand, it is known that rapids areas harbor their own specialized and commonly endemic ichthyofaunas, adapted to life in turbulent water environments (e.g., Hora, 1930; Roberts and Stewart, 1976; Kullander, 1988; Isbrücker and Nijssen, 1991; Jégu, 1992, 2004; Zuanon, 1999; Flausino Junior et al., 2016; Collins et al., 2018; Fitzgerald et al., 2018; Machado et al., 2018). Studies of natural history and ecology focusing on associations of fish species in rapids and waterfalls are rare (Balon, 1974; Balon and Stewart, 1983; Casatti and Castro, 1998; Fitzgerald et al., 2018). Mainly due to sampling difficulties, little is known about the way of life and structure of fish communities in these environments. Most of the information on the fish assemblages of rapids in the Amazon region is concentrated in unpublished technical reports, especially those related to studies of environmental impacts of proposed hydroelectric projects. In addition, existing information is usually restricted to occurrence records based on poorly resolved taxonomy, with data obtained without standardized sampling/experimental design among studies, making data interpretation and quantitative comparisons difficult. In Brazil, publications of rheophilic communities are restricted to a natural history study of rheophilic fish fauna of a stream in the headwaters of the São Francisco River (Casatti and Castro, 1998), a study (Fitzgerald et al., 2018) and an unpublished doctoral thesis (Zuanon, 1999) focusing on the Volta Grande region of the Xingu River, and a study of the fishes associated with podostomacean mats in the Aripuanã River (Flausino Junior et al., 2016). Despite the scarcity of studies, it is known that rheophilic environments harbor species–rich and highly diverse assemblages, comprised of many endemics.

Dearth of studies of rheophilic taxa is in part a consequence of the still poor general knowledge of distribution of freshwater biodiversity (Vörösmarty et al., 2010). In the Neotropics, this impediment is the result of severe undersampling of freshwater taxa in general (Lundberg et al., 2000; Lévêque et al., 2007; Alofs et al., 2014), leading to many taxa not being evaluated by the IUCN or national conservation agencies, or when evaluated being listed as data deficient (Collen et al., 2014). This, in turn, hampers prioritizing freshwater habitats for conservation (Abell, 2002; Darwall et al., 2011; Frederico et al., 2016, 2018).

Perhaps the most emblematic groups of fishes occupying rheophilic habitats in the Neotropics are the suckermouth armored catfishes (Loricariidae). The loricariids have acquired spectacular adaptations in their mouth, labial and dental morphologies that allow them to occupy rheophilic habitats and diversify within them (Lujan et al., 2012, 2017; Roxo et al., 2017; Collins et al., 2018). They probably are the most strictly associated group of fishes with rheophilic habitats (Reis et al., 2003). However, the loricariids are not the only group to have colonized and diversified within rheophilic habitats. Several clades of the plant eating pacus (Serrasalmidae) are also rheophilic specialists (e.g., Jégu, 2004; Andrade et al., 2016, 2017; Machado et al., 2018), as are some cichlid (Cichlidae) (e.g., Kullander, 1988) and anostomid (Anostomidae) (e.g., Santos and Jégu, 1987) species.

Among–river discontinuity of rheophilic habitats causes high degree of endemism, with few species occupying more than a single river basin (Reis et al., 2003), and those that occur in more than one river generally represent divergent, independently evolving lineages (Collins et al., 2018). By the same token, discontinuous rheophilic habitats within the same river system could lead to structuring of rheophilic fish species. Rheophilic habitats are islands of suitable habitat within a waterscape of unsuitable habitat, analogous to the rock islands of the great African lakes (Salzburger et al., 2014). It is with this objective that we decided to study to what degree, if any, the rheophilic fish fauna occupying distinct and unconnected rheophilic habitat is genetically structured along the Araguaia River. The implication of population structuring of rheophilic fishes, or the occupation of different rapids by different lineages would make reophilic communities occupying different rapids even more distinct and non–substitutable when planning environmental mitigatory measures.

For this purpose we chose to sample and analyze the less well studied cichlid and anostomiid species rather than the obvious candidate loricariid species. If the rheophilic habitats really are analogous to the rocky islands and outcrops in the great African lakes, then we should observe significant amount of population structuring between rheophilic cichlids and anostomiids occupying different rapids along the river.

## Materials and methods

### Field sampling

**Figure 1:**
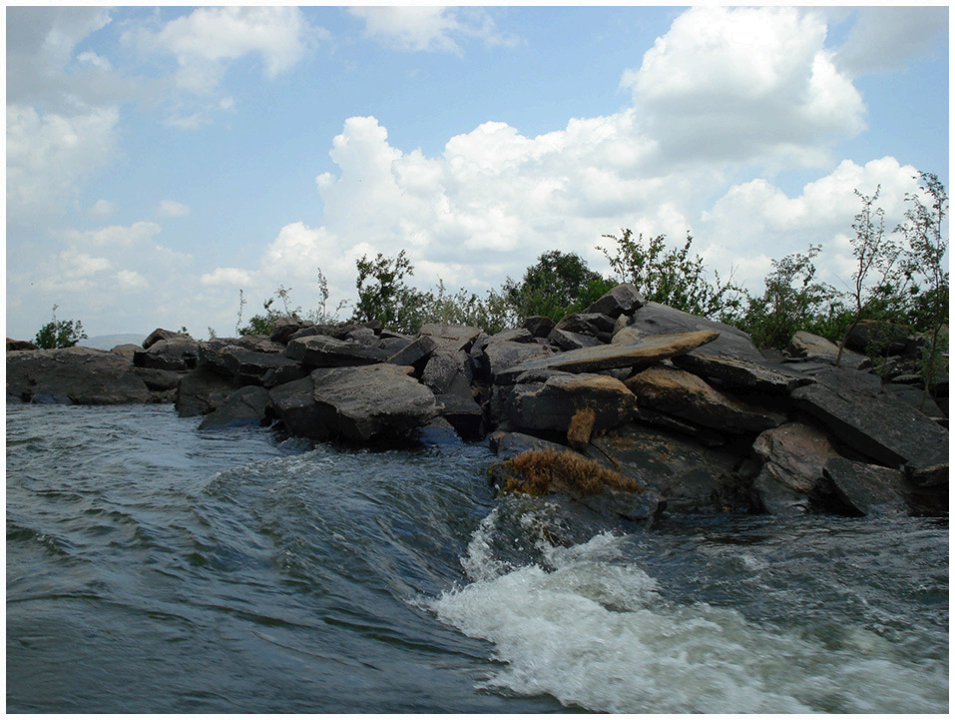
A close up of an island within the Santa Isabel rapid complex showing habitat structure and flowering Podostemaceae.

**Figure 2:**
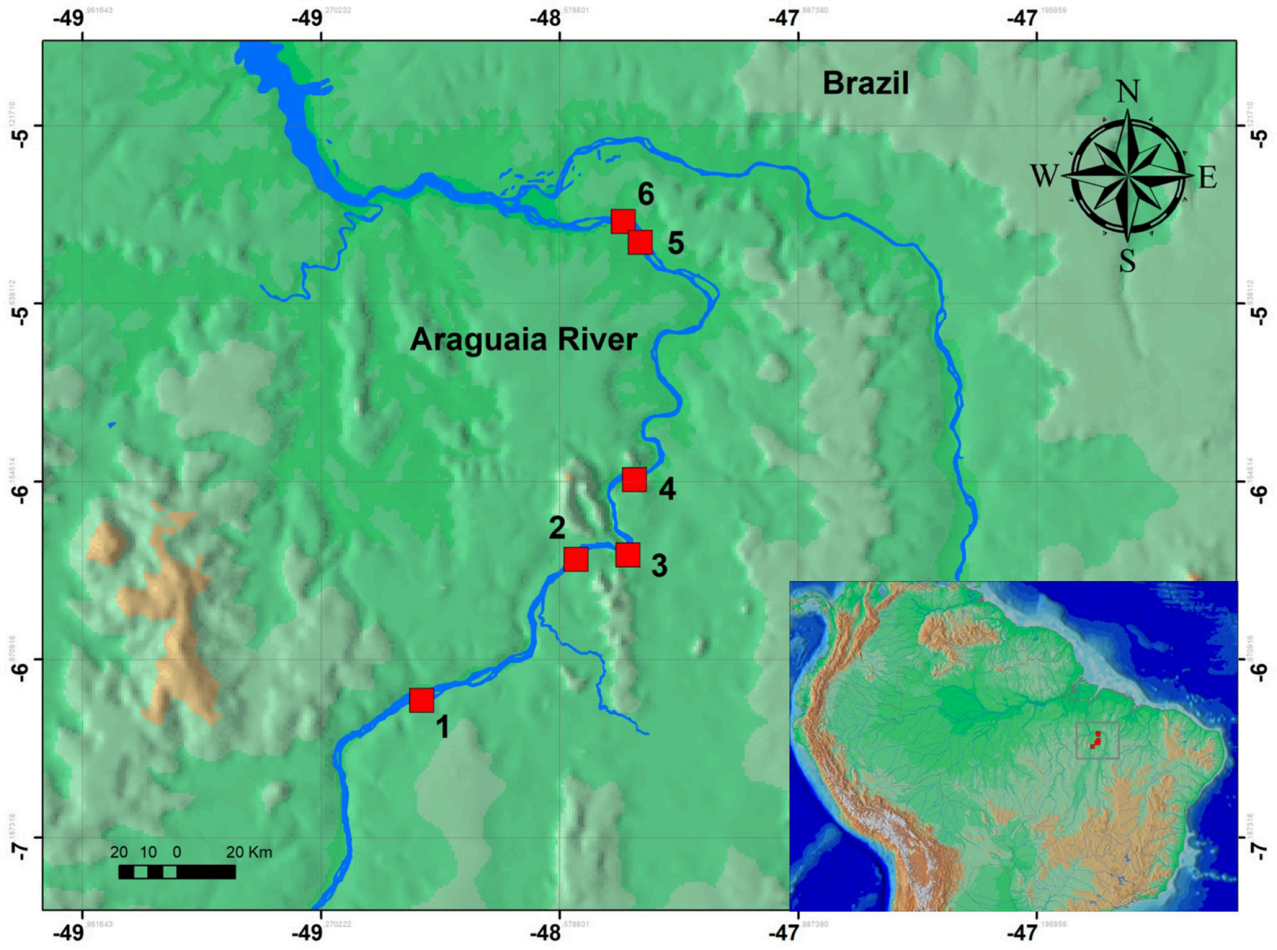
Map of the six regions of rapids sampled in this study. Localities are: (1) Zé dos Gatos rapid; (2) São Miguel rapid; (3) Remanso dos Botos rapid; (4) Santa Isabel rapid; (5) São Bento rapid; (6) Rebojo rapid.

Over an eight day period, we sampled six stretches of rapids in the lower Araguaia River (Table 1; Figure 1). The sampled rapids were Cachoeira do Zé dos Gatos, São Miguel, Remanso dos Botos, Santa Isabel, São Bento, and Rebojo (Figure 2). Distance between the Cachoeira do Zé dos Gatos and Rebojo rapids is 150 km, while the São Bento and Rebojo rapids are separated by less than 1 km.

**Table 1:** Location of sampled rapids in the Araguaia River.

**Locality**	**State**	**Municipality**	**Date**	**Latitude**	**Longitude**
Zé dos Gatos rapid	Pará	Piçarra	8–9/sept/09	−6.78927	−48.97752
Santa Isabel rapid	Pará	Santa Isabel	10–11/sept/09	−6.14894	−48.36074
São Miguel rapid	Tocantins	Xambioá	12/sept/09	−6.38009	−48.53014
Remanso dos Botos rapid	Tocantins	Xambioá	13/sept/09	−6.36741	−48.37892
São Bento rapid	Tocantins	Araguatins	14/sept/09	−5.46001	−48.34394
Rebojo rapid	Tocantins	Araguatins	15/sept/09	−5.39970	−48.39315

After a study of the prevailing conditions in the field, we decided to divide the team into a group of two people collecting fish with gilnets (24–60 mm mesh between opposing nodes) and castnets (10 mm mesh between opposing nodes) and one person catching fish with line and hook. This strategy aimed to optimize capture effort and to sample the available diversity of habitats occupied by anostomids and cichlids. Sampling time at each site was approximately 8 h with the the exception of the Cachoeira do Zé dos Gatos and Santa Isabel rapids where we spent approximately 16 h each.

We focused on sampling two groups: cichlids which generally are sedentary and some species are rheophilic specialists, and anostomids which also have rheophilic specialists but as a group may be considered vagil. Final choice of species included in this study was conditioned by our ability to obtain sufficient number of specimens from at least four of the six rapids.

Preliminary identification of specimens was carried out in the field. Final identification of the collected specimens was made based on comparisons with samples deposited in the INPA Fish Collection (Manaus, AM), consultation of original descriptions and use of published dichotomous keys (when available).

All field collections were authorized by IBAMA/SISBIO 11325-1, and access to genetic resources was authorized by permit No. 034/2005/IBAMA.

### DNA extraction, molecular markers and data pre-processing

DNA extraction was performed using standard phenol/chloroform protocol (Sambrook and Russell, 2001). For the molecular analyzes we chose to amplify the control region *CR* of mitochondrial DNA (mtDNA), since it is the fastest evolving section of the mitochondrial genome, and thus is most likely to register fine-scale population structuring (Meyer, 1993). We obtained approximately 650–750 base pairs for each individual. We analyzed the same region of mtDNA for all individuals and thus eliminated any confounding effects of using different mtDNA gene regions with potentially different rates of molecular evolution in different species.

A common forward primer was used for all species (ProF: 5′-AACYCCCRCCCCTAACYCCCAAAG-3′). For anostomids we used the reverse primer DLOstariR.1 (5′-gtaaaacgacggccagTCCTGGTTTHGGGGTTTRAC6AG-3′), while for cichlids we used the reverse primer DLPercoR.1 (5′-gtaaaacgacggccagTCCTGTTTCCGGGGGGTTTACAG-3′). Both reverse primers incorporated an M13(-21) tail on their 5′ end. The 15 μL PCR mix included 1.2 μL of 10 mM dNTPs (2.5 mM each DNTP), 1.5 μL 10× buffer (75 mM Tris HCL, 50 mM KCL, 20 mM (NH_4_)_2_SO_4_), 1.2 μL 25 mM MgCl_2_, 1.5 μL of primer cocktails (2 pmol each), 0.5 μL of Taq DNA polymerase, 1 μL of template DNA and 6.6 μL ddH_2_O. PCR conditions were: 94°C (30 s); 35 cycles of 94°C (30 s), 50°C (35 s), 68°C (90 s); followed by 68°C (5 min).

PCR products were evaluated on a 1% percent agarose gel, and then purified using Exo-SAP (Exonuclease—Shrimp Alcaline Phosphatase) following the manufacturer's suggested protocol (Werle et al., 1994). The control region products were sequenced using the M13(-21) primer. Sequencing reactions were carried out according to the manufacturer's recommendation for the ABI BigDye Terminator cycle sequencing mix, using an annealing temperature of 50°C (Platt et al., 2007). Sequencing reactions were precipitated using standard EDTA/EtOH protocol, and was resolved on the ABI 3130xl (Life Technologies) automatic sequencer. Sequence products were edited, concatenated and aligned using the Clustal W algorithm (Thompson et al., 1994) followed by manual adjustments as implemented in the program Genious v8.1.7 (Kearse et al., 2012). Sequences are deposited in GenBank under accession numbers MH514035–MH514287.

### Analyses

Using the mtDNA control region we tested whether the populations of the species found at different rapids are differentiated from one another. We tested this hypothesis using the Analysis of Molecular Variance (AMOVA) (Excoffier et al., 1992) as implemented in the program Arlequin v3.5 (Excoffier and Lischer, 2010). Significant differentiation was interpreted as lack of gene exchange between populations of individuals of the same species found in different rapids.

We also delimited lineages using the Bayesian approach implemented in the program BAPS v6.0 (Corander et al., 2008). Individual level mixture analysis was performed for different maximum number of lineages (*k* = 1–5), with 10 independent runs for each value of k. The k with the highest posterior probability was selected as representing the correct data partition. We subsequently tested for a non-random distribution of lineages across the different rapids from which these lineages were sampled using the Fisher's exact test as implemented in the R statistical language (R Development Core Team, 2011).

Haplotype network and geographic occurrence of haplotypes was estimated in the program HapView (Salzburger et al., 2011) using a phylogeny of haplotypes generated in RAxML v8.2.0 (Stamatakis, 2014).

Pairwise uncorrected p–distances were calculated between all individuals of a given species using Genious v8.1.7 (Kearse et al., 2012).

Finally, we calculated the Tajima's D (Tajima, 1989) for each species.

All analyses were carried out separately for each species, assuming α = 0.05.

## Results

**Figure 3:**
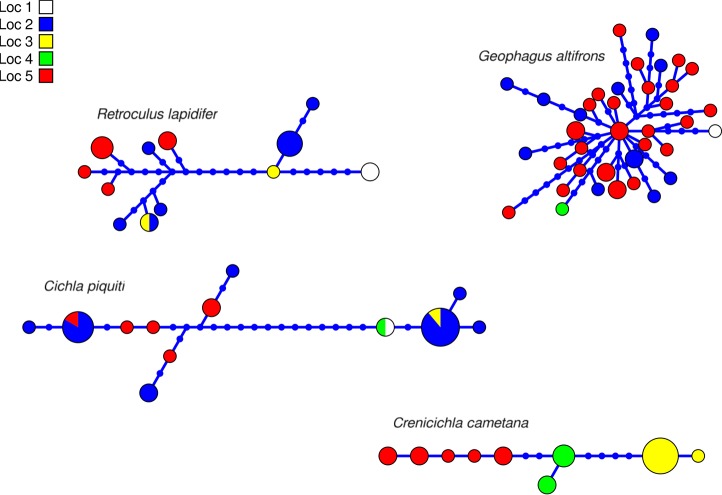
Haplotype network of four species of *Cichlidae* indicating geographic occurance of the haplotypes. Networks were generated in hapview, and haplotype size is proportional among networks and to the number of individuals with that haplotype. Localities are: (1) Zé dos Gatos rapid; (2) São Miguel rapid; (3) Remanso dos Botos rapid; (4) Santa Isabel rapid; (5) São Bento rapid and Rebojo rapid.

**Figure 4:**
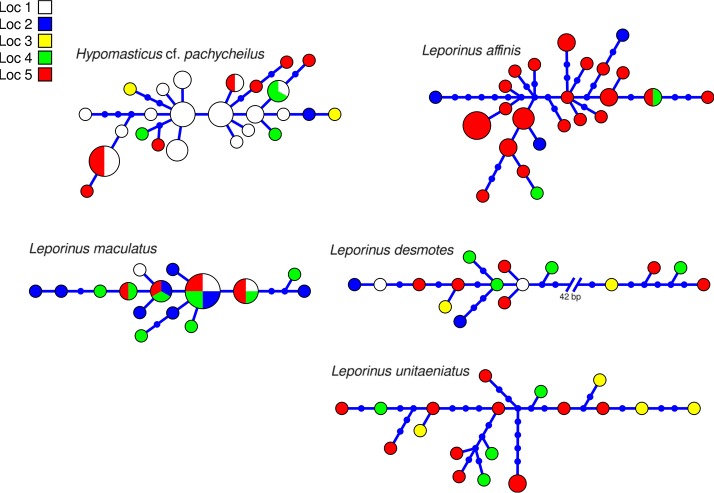
Haplotype network of five species of *Anostomidae* indicating geographic occurance of the haplotypes. Networks were generated in hapview, and haplotype size is proportional among networks and to the number of individuals with that haplotype. Localities are: (1) Zé dos Gatos rapid; (2) São Miguel rapid; (3) Remanso dos Botos rapid; (4) Santa Isabel rapid; (5) São Bento rapid and Rebojo rapid.

**Figure 5:**
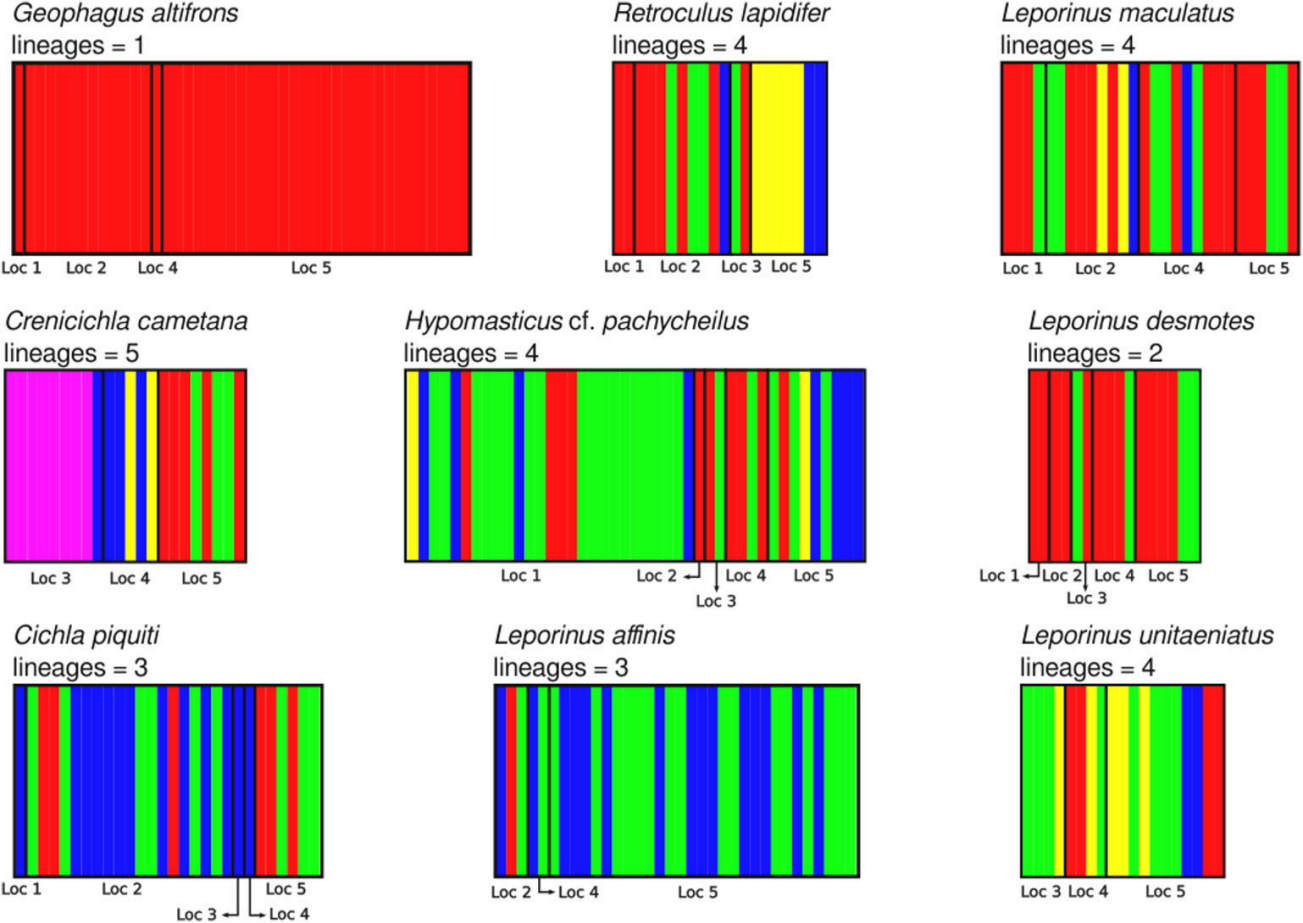
Identification of lineages of all nine species analyzed in this study using BAPS v6.0. Localities are: (1) Zé dos Gatos rapid; (2) São Miguel rapid; (3) Remanso dos Botos rapid; (4) Santa Isabel rapid; (5) São Bento rapid and Rebojo rapid.

The aligned DNA data matrix comprised nine taxa, 253 individuals by 640–778 bp. Number of individuals per species varied from 16 to 43 (Table 2). Eight of the nine taxa had more than one lineage, with upto five lineages being observed in *Crenicichla cametana*. In total we observed 175 unique haplotypes (Figures 3, 4) grouped into 29 lineages (Figure 5). The existence of multiple lineages within rheophilic taxa and *Leporinus desmotes* is further reforced by significantly positive Tajima's D metrics. Significant population structuring was observed in five of the nine species analyzed, and non-randon association of lineages and rapids was observed in three of the eight species with multiple lineages. Results of tests of among-rapids population differentiation, Tajima's D and Fu's Fs, and non-random distribution of lineages among rapids is summarized in Tables 3, 4, respectively.

**Table 2:** Number of individuals of each species analyzed from the rapids of the Araguaia River.

**Species**	**Zé dos Gatos**	**Santa Isabel**	**São Miguel**	**Remanso dos Botos**	**São Bento & Rebojo**	**Total**
*Retroculus lapidifer*	2	9	2	0	7	20
*Geophagus altifrons*	1	12	0	1	29	43
*Cichla piquiti*	1	19	1	1	6	28
*Crenicichla cametana*	0	0	9	5	8	22
*Hypomasticus *cf*. pachycheilus*	27	1	2	4	9	43
*Leporinus maculatus*	4	9	0	9	6	28
*Leporinus affinis*	0	3	0	2	29	34
*Leporinus desmotes*	2	2	2	4	6	16
*Leporinus unitaeniatus*	4	4	0	0	11	19

**Table 3:** Analysis of population differentiation among rapids of the Araguaia River, and demography of each species.

**Species**	**ϕ_*ST*_**	**AMOVA**	**Tajima's D**
**Cichlidae**			
*Retroculus lapidifer*	0.35119	*p* = 0.00040 ± 0.00019	0.53176^†^
*Geophagus altifrons*	0.11732	*p* = 0.00802 ± 0.00086	−1.79152^*^
*Cichla piquiti*	0.29184	*p* = 0.01050 ± 0.00096	2.35861^‡^
*Crenicichla cameta*	0.86498	*p* < 0.00001	1.57836^‡^
**Anostomidae**			
*Hypomasticus *cf*. pachycheilus*	0.15674	*p* = 0.00406 ± 0.00066	−0.80127
*Leporinus maculatus*	−0.04735	*p* = 0.87851 ± 0.00363	−1.04746
*Leporinus affinis*	0.00075	*p* = 0.43525 ± 0.00483	−1.09098
*Leporinus desmotes*	−0.19958	*p* = 0.95950 ± 0.00191	0.96519^†^
*Leporinus unitaeniatus*	0.02545	*p* = 0.29604 ± 0.00478	−0.19675

* ,significantly negative;

† ,positive;

‡ *,significantly positive*.

**Table 4:** Analysis of non-random distribution of lineages among rapids of the Araguaia River.

**Species**	**# of lineages**	**Fisher's exact test**	**lineage p–distance (%)**
**Cichlidae**			
*Retroculus lapidifer*	4	*p* = 0.00100	2.24
*Geophagus altifrons*	1	NA	1.46
*Cichla piquiti*	3	*p* = 0.08546	2.58
*Crenicichla cameta*	5	*p* = 0.00050	1.57
**Anostomidae**			
*Hypomasticus *cf*. pachycheilus*	4	*p* = 0.00700	1.04
*Leporinus maculatus*	4	*p* = 0.94250	0.89
*Leporinus affinis*	3	*p* = 0.16490	1.50
*Leporinus desmotes*	2	*p* = 1	7.33
*Leporinus unitaeniatus*	4	*p* = 0.71860	1.54

### Species accounts

#### *Retroculus lapidifer* (cichlidae) (Figure 6A)

This species is a sand-sifter and a rheophilic specialist. The collections of this species occurred in the localities of Cachoeira do Zé dos Gatos (*N* = 2), Santa Isabel (*N* = 9), São Miguel (*N* = 2) and Rebojo and São Bento (*N* = 7). Within this species we observe at least four genetic lineages with up to 2.24% p–distance sequence divergence. Analysis of Molecular Variance (AMOVA) indicated high levels of geographic structuring (ϕ_*ST*_ = 0.35119; *p* = 0.00040 ± 0.00019). Tajima's D was positive (0.53176). The hypothesis of random associations of genetic lineages with rapids was rejected (Fisher's exact test, *p* = 0.00100). These lineages were largely restricted to specific rapids.

**Figure 6:**
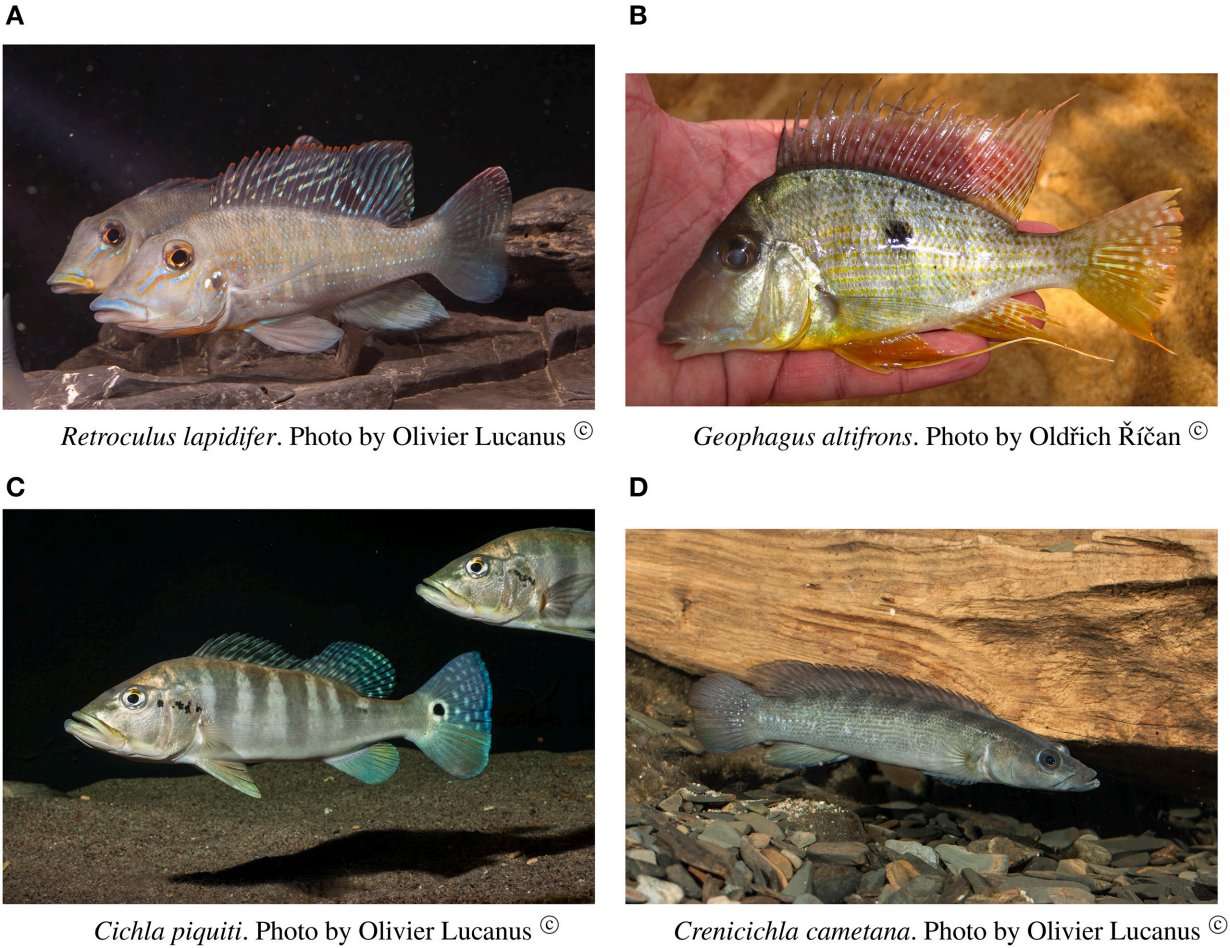
Cichlid species analyzed in this study.

#### *Geophagus altifrons* (cichlidae) (Figure 6B)

This species is a habitat generalist but is commonly found in shallow sandy areas of stretches of rapids. The collections of this species occurred in the localities of Cachoeira do Zé dos Gatos (*N* = 1), Santa Isabel (*N* = 12), Remanso dos Botos (*N* = 1) and Rebojo and São Bento (*N* = 29). We observed only one genetic lineage within this species. AMOVA indicated geographic structuring (ϕ_*ST*_ = 0.11732; *p* = 0.00802 ± 0.00086). Tajima's D was negative and significant (−1.79152).

#### *Cichla piquiti* (cichlidae) (Figure 6C)

This species is usually found in structured habitats, including rapids, where it lurks and waits for its prey. The collections of this species occurred in the localities of Cachoeira do Zé dos Gatos (*N* = 1), Santa Isabel (*N* = 19), São Miguel (*N* = 1), Remanso dos Botos (*N* = 1) and Rebojo and São Bento (*N* = 6). We observed three genetic lineages with up to 2.58% p–distance sequence divergence. AMOVA indicated high levels of geographic structuring (ϕ_*ST*_ = 0.29184; *p* = 0.01050 ± 0.00096). Tajima's D was positive and significant (2.35861). The hypothesis of random associations of genetic lineages with rapids was not rejected (Fisher's exact test, *p* = 0.08546).

#### *Crenicichla cametana* (cichlidae) (Figure 6D)

This species is a relatively small ambush predator, and a specialist of rapids. The collections of this species occurred in the localities of São Miguel (*N* = 9), Remanso dos Botos (*N* = 5) and Rebojo and São Bento (*N* = 8). We observed five divergent genetic lineages with up to 1.57% p–distance sequence divergence. AMOVA indicated high levels of geographic structuring (ϕ_*ST*_ = 0.86498; *p* < 0.00001). Tajima's D was positive and significant (1.57836). The hypothesis of random association of genetic lineages with rapids was rejected (Fisher's exact test, *p* = 0.00050). These lineages were largely restricted to specific rapids.

#### Hypomasticus cf. *pachycheilus* (anostomidae) (Figure 7A)

This species is a rheophilic specialist, an algae scraper, found in fast flowing waters of rapids. The collections of this species occurred in the localities of Cachoeira do Zé dos Gatos (*N* = 27), Santa Isabel (*N* = 1), São Miguel (*N* = 2), Remanso dos Botos (*N* = 4) and Rebojo and São Bento (*N* = 9). We observed four genetic lineages with up to 1.04% p–distance sequence divergence. AMOVA indicated geographic structuring (ϕ_*ST*_ = 0.15674; *p* = 0.00406 ± 0.00066). Tajima's D was negative (-0.80127). The hypothesis of random association of genetic lineages with rapids was rejected (Fisher's exact test, *p* = 0.00700).

**Figure 7:**
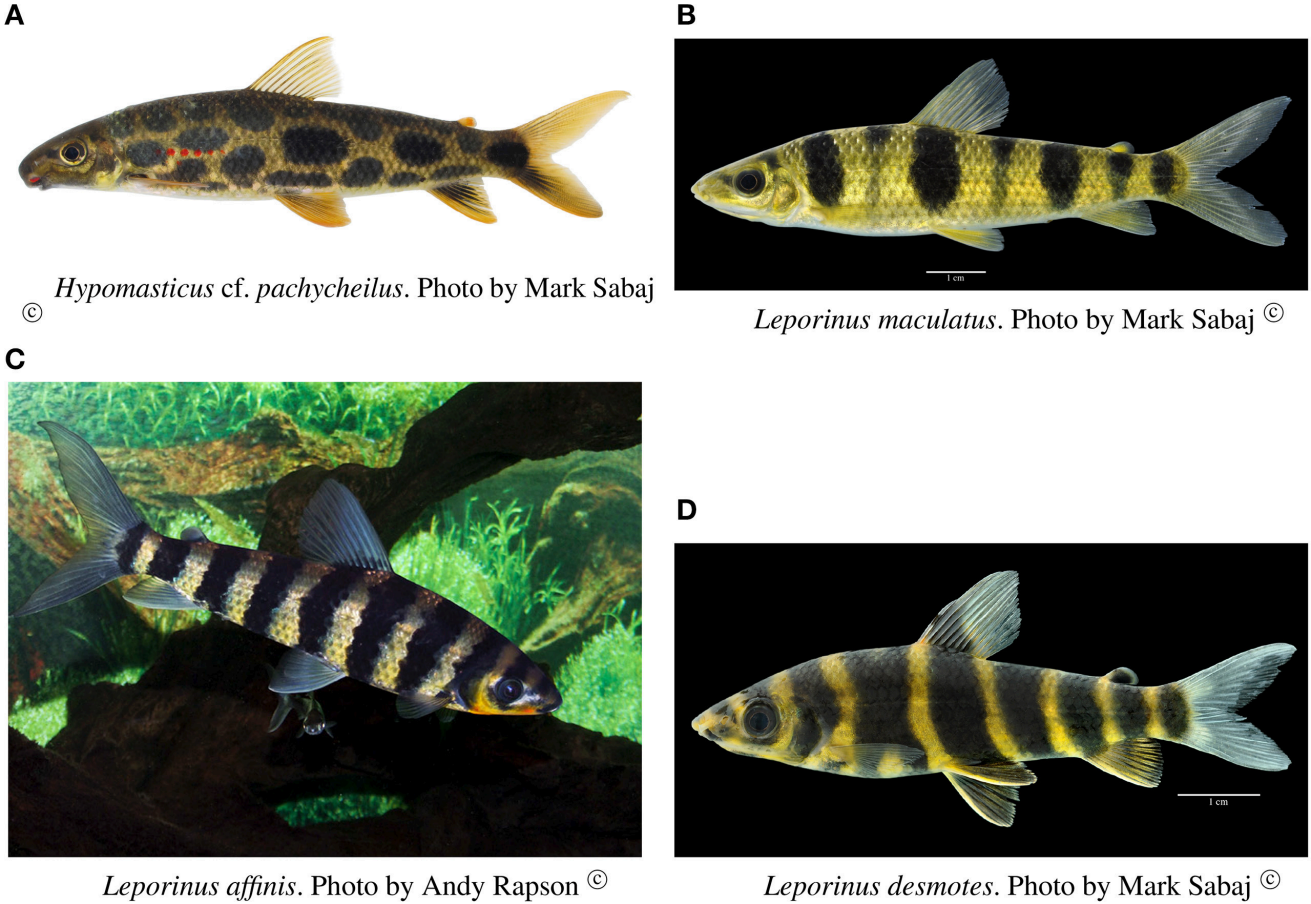
Anostomid species analyzed in this study.

#### *Leporinus maculatus* (anostomidae) (Figure 7B)

This species is found in relatively slow and fast water regions of rapids; although it is not a rapids specialist, shows a strong association with rocky substrates. The collections of this species occurred in the localities of Cachoeira do Zé dos Gatos (*N* = 4), Santa Isabel (*N* = 9), Remanso dos Botos (*N* = 9) and Rebojo and São Bento (*N* = 6). We observed four genetic lineages with up to 0.89% p–distance sequence divergence. AMOVA indicated no genetic structuring in the species (ϕ_*ST*_ = −0.04735; *p* = 0.87851 ± 0.00363). Tajima's D was negative (−1.04746). The hypothesis of random association of lineages with the rapids was not rejected (Fisher's exact test, *p* = 0.94250).

#### *Leporinus affinis* (anostomidae) (Figure 7C)

This species is a habitat generalist that is found in both slow and fast water regions of rapids, and also in a wide variety of habitats and water flows within the Araguaia-Tocantins river system. The collections of this species occurred in the localities of Santa Isabel (*N* = 3), Remanso dos Botos (*N* = 2) and Rebojo and São Bento (*N* = 29). We observed three divergent genetic lineages with up to 1.50% p–distance sequence divergence. AMOVA indicated absence of geographic structuring (ϕ_*ST*_ = 0.00075; *p* = 0.43525 ± 0.00483). Tajima's D was negative (−1.09098). The hypothesis of random association of lineages with the rapids was not rejected (Fisher's exact test, *p* = 0.16490).

#### *Leporinus desmotes* (anostomidae) (Figure 7D)

This species is found in both slow and fast water regions of rapids; although it is not a rapids specialist, it shows strong association with rocky substrates. The collections of this species occurred in the localities of Cachoeira do Zé dos Gatos (*N* = 2), Santa Isabel (*N* = 2), São Miguel (*N* = 2), Remanso dos Botos (*N* = 4) and Rebojo and São Bento (*N* = 6). We observed two divergent genetic lineages with upto 7.33% p–distance sequence divergence. AMOVA indicated lack of genetic structuring (ϕ_*ST*_ = −0.19958; *p* = 0.95950 ± 0.00191). Tajima's D was positive (0.96519). The hypothesis of random association of lineages with the rapids was not rejected (Fisher's exact test, *p* = 1.00).

#### *Leporinus unitaeniatus* (anostomidae)

This species is found in both slow and fast water regions of rapids, and although it is not a rapids specialist, it is a rocky substrate dweller. The collections of this species occurred at the localities of Cachoeira do Zé dos Gatos (*N* = 4), Santa Isabel (*N* = 4), Remanso dos Botos (*N* = 0) and Rebojo and São Bento (*N* = 11). We observed two divergent genetic lineages with upto 1.54% p–distance sequence divergence. AMOVA indicated lack of genetic structuring (ϕ_*ST*_ = 0.02545; *p* = 0.29604 ± 0.00478). Tajima's D was negative (−0.19675). The hypothesis of random association of lineages with the rapids was not rejected (Fisher's exact test, *p* = 0.71860).

## Discussion

In this study we have shown an unprecedented level of fine-scale population structuring among fish species that occupy rapids stretches of a large Amazonian river. Five of the nine studied species showed either non–random distribution of genetic diversity among rapids, non–random distribution of lineages among rapids or both. Among these, three rheophilic specialists (the cichlids *Retroculus lapidifer* and *Crenicichla cametana* and the anostomid *Hypomasticus cf. pachycheilus*) show strong population level differentiation between rapids and non–random distribution of lineages among rapids. The remaining two species of cichlids (*Geophagus altifrons* and *Cichla piquiti*) are not rapids specialists but both are phylopatric and sedentary species which favors the occurrence of genetic structuring in habitat patches along the river. Geneflow among populations of these species occupying distinct rapids is low, in all five instances *Nm* < 2 and in three instances *Nm* < 1. In addition to the nine analyzed species, two additional species of rheophilic anostomids, *Leporellus vittatus* and *Leporinus bistriatus*, probably exhibit a similar pattern of genetic differentiation as *Hypomasticus cf. pachycheilus*. However, this could not be tested because of the low sample size resulting from the difficulty in collecting samples. Finally, *Leporinus desmotes* was comprised of two deeply divergent—7.33% p–distance—lineages that may represent two cryptic species. These results parallel those of the only other fine–scaled population study by Markert et al. (2010) of two rheophilic cichlid fish species collected at five rapids–associated sites of the lower Congo River separated by no more than 100 km. Therefore, significant levels of within river system geographic structuring is likely to be the norm for rheophilic fish fauna inhabiting rapids of not just the Araguaia, but all Amazonian rivers.

In summary, rheophilic and sedentary species such as cichlids showed high levels of population differentiation among rapids, and consequently low levels of geneflow, in some cases sufficiently low to prevent divergence of these populations by genetic drift. These species were also comprised of distinct lineages non–randomly distributed among rapids. When non-random distribution of lineages was observed, in many cases these lineages were allopatric, i.e., there was no overlap in the distribution of these lineages. This signifies that there are some lineages that only occur in specific rapids and/or groups of rapids, i.e., they are micro-endemic. However, none of these lineages are readily identifiable using traditional external morphological traits.

The occurrence of such remarkable population structuring of rheophilic fishes in the rapids along the Araguaia River points to a possible high level of extinction risk for a considerable portion of the Amazon fish fauna, at an unexpected spatial scale. The Amazon River, as other major rivers, is rapidly being developed and its hydroelectric potential is starting to be realized (Lees et al., 2016; Winemiller et al., 2016). However, more so than any other major river, the Amazon and its affluents have been largely unimpacted by large–scale anthropogenic actions and flow through predominantly pristine landscapes (Winemiller et al., 2016). Most of the basin's hydroelectric potential has also been largely untapped, but there is strong pressure to do so both in Brazil as well as other Amazonian countries (Finer and Jenkins, 2012; Fearnside, 2016). At the moment hydroelectric projects have concentrated in regions with very large energy generating potential—e.g., Tucurui or Belo Monte supplying electricity to energy intensive industries—or on those supplying local needs—e.g., Balbina supplying electricity to Manaus and Curua-una supplying electricity to Santarém, both in the Brazilian Amazon.

Dam construction is subject to environmental impact studies which generally consist of faunal and floral surveys. More complex biological processes acting beyond the seasonal scale are generally not addressed by time constraints of the licencing process. Part of the licensing process requires a plan for the mitigation of environmental disturbances caused by the construction and functioning of the hydoelectric complex, although it is questionable to what extent, if any, this goal can be accomplished (Fearnside, 2012). Rarely are molecular tools employed, and when they are, they do not necessarily address relevant conservation–driven questions. If molecular tools are used, they are predominantly employed to test to what extent, if any, the area of rapids functions as barriers to geneflow to commercially important species with distributions upstream and downstream of the rapids. The presumed motivation is to determine if fish ladders should be constructed or not. The effectiveness of fish ladders in Amazonian context is questionable, however (Pelicice and Agostinho, 2008). In the only case in the Brazilian Amazon where fish ladders were constructed and their effectiveness evaluated—the Santo Antônio hydroelectric complex on the upper Madeira River—many of the migratory species, including the goliath catfishes (*Brachyplatystoma* spp.), that used to migrate upstream of the Teôtonio rapids for reproduction scarcely do so any more, while species previously isolated by the rapids have used the ladders to invade upstream (Cella-Ribeiro et al., 2017).

Molecular tools are not, however, used to evaluate the effects on rheophilic fauna and flora, and consequently to what extent, if any, can the destruction of this fauna and flora can be compensated with mitigatory measures. To our knowledge this study is the first to attempt to specifically address this question.

### Implication for conservation

Rapids–dwelling fishes have ecological specializations uniquely linked to the occupation of these habitats. One of these specializations is the use of podostemaceous plants as a place of shelter, growth, foraging and direct consumption by herbivorous species (Flausino Junior et al., 2016). As an example, strongly rheophilic pacus species (Characiformes: Serrasalmidae), such as those of the genera *Mylesinus* and *Tometes*, present cutting teeth used in leaf pruning of these plants (e.g., Jégu and Santos, 1988; Santos et al., 1997; Vitorino Júnior et al., 2016; Andrade et al., 2017). The strong dependence on podostemaceous mats as food makes these pacus particularly vulnerable to the environmental impacts resulting from dam construction. The reduction of densities and numbers of the Podostemaceae in rivers with artificially regulated flow seems to have caused a significant population reduction of the pacu *Mylesinus paraschomburgi*, a specialized consumer of these plants (Santos et al., 1997). Similar responses to environmental perturbations have been observed in other rheophilic fish species, such as the anostomids (Santos et al., 1997), loricariids (Zuanon, 1999) and cichlids (Kullander, 1988, 1991; Zuanon, 1999).

In addition to the Podostemaceae, many rapids–dwelling fish species use epiliton, a layer of organic debris, algae and small invertebrates that cover the rocky substratum in the rapids, as food. There is evidence that rheophilic fishes are strongly dependent on this type of food (Casatti and Castro, 1998; Zuanon, 1999), and environmental impacts that alter the quality and quantity of this resource negatively affect the local ichthyofauna, with loss of species and trophic relationships.

In addition to the close and direct ecological relationship between fishes and rapids, the spatial distribution of rheophilic habitats within a course of a river also contributes to the fragility of the system. In many rivers, rapids are environments distributed in a mosaic, isolated from each other by other habitat types unsuitable for rheophilic fauna and flora. Not only are rheophilic habitats characterized by high levels of endemism and taxonomic diversity, our study suggests that rheophilic habitats are also characterized by high levels of populational structuring and phylogenetic diversity. Thus while fish assemblages and communities may be similar among the different rapids of a same river, our study indicates that they are composed of different lineages, and in some cases even ecologically equivalent but morphologically cryptic species.

Areas of rapids of several Amazonian rivers have already been radically altered by the construction of hydroelectric dams. In all these cases, the rapids and much of the associated ichthyofauna disappeared (Agostinho et al., 2008), and based on our study, this fauna is not substituitable by fauna occuring outside the area of direct impact of the hydroelectric dam. The construction of reservoirs causes a permanent change of lotic to lentic habitats, ensuing in the local extinction of the specialized rheophilic fauna and flora whose habitat became submerged under the hydroelectric reservoir. The elevated levels of phylogenetic diversity, the non-random distribution of this diversity among the rapids, with lineages often being restricted to a specific rapid or groups of rapids, implies that the disappearance of the rheophilic ichthyofauna from areas of hydroelectric projects probably leads to the extiction of these lineages, even if other lineages survive elsewhere.

#### We may ask: why does this matters?

Although ecologically equivalent lineages may persist in other areas, we are loosing unique and singular evolutionary heritage. Once extinct, these lineages can never become “unextinct.” Even more importantly, these lineages are an inevitable consequence of evolution, and thus are intricately linked to the underlying evolutionary processes that gave rise to them. By destroying these lineages, we, inevitable, impair or even destroy our potential to understand these processes and the origins of this amazing biodiversity.

An argument may also be made that we should not focus on lineages, but rather on species, since species are the fundamental units of biodiversity (Soulé and Wilcox, 1980). However, species are lineages (Simpson, 1961; de Queiroz, 2007), and need to be understood in this light. If we treat species as classes, or do not recognize that species may be morphologically cryptic, often relying on incomplete taxonomic knowledge, we limit ourselves in what we can understand of the evolutionary history and the evolutionary potential of the studied groups.

Ultimately we must recognize the lineage-like nature of species and focus our studies on them if we hope to understand the evolution and maintenance of biodiversity (Willis, 2017), and if we hope to minimize our contribution to the sixth mass biological extinction (Ceballos et al., 2015).

## Author contributions

TH, IF, and JZ conceived the experiment and obtained funding. TH, NM, and JZ conducted fieldwork and collected specimens. JZ morphotyped and revised specimens. NM and TH collected molecular data. TH analyzed the results. TH wrote the manuscript with substantial contributions from JZ. All authors contributed to and reviewed the manuscript.

### Conflict of interest statement

The authors declare that the research was conducted in the absence of any commercial or financial relationships that could be construed as a potential conflict of interest.

## References

[B1] Abell R. (2002). Conservation biology for the biodiversity crisis: a freshwater follow-up. Conserv. Biol. 16, 1435–1437. 10.1046/j.1523-1739.2002.01532.x 35701979

[B2] Agostinho Â. A. Pelicice F. M. Gomes L. C. (2008). Dams and the fish fauna of the Neotropical region: impacts and management related to diversity and fisheries. Braz. J. Biol. 68 4 Suppl:1119–1132. 10.1590/S1519-69842008000500019 19197482

[B3] Alofs K. M. Liverpool E. A. Taphorn D. C. Bernard C. R. López-Fernández H. (2014). Mind the (information) gap: the importance of exploration and discovery for assessing conservation priorities for freshwater fish. Divers. Distribut. 20, 107–113. 10.1111/ddi.12127

[B4] Andrade M. C. Machado V. N. Jégu M. Farias I. P. Giarrizzo T. (2017). A new species of *Tometes* Valenciennes 1850 (Characiformes: Serrasalmidae) from Tocantins–Araguaia River Basin based on integrative analysis of molecular and morphological data. PLoS ONE 12:e0170053. 10.1371/journal.pone.0170053 28422969 PMC5396854

[B5] Andrade M. C. Sousa L. M. Ota R. P. Jégu M. Giarrizzo T. (2016). Redescription and geographical distribution of the endangered fish *Ossubtus xinguense* Jégu 1992 (Characiformes, Serrasalmidae) with comments on conservation of the rheophilic fauna of the Xingu River. PLoS ONE 11:e0161398. 10.1371/journal.pone.0161398 27662358 PMC5035070

[B6] Balon E. K. (1974). Fishes from the edge of Victoria Falls, Africa: demise of a physical barrier for downstream invasions. Copeia 1974, 643–660. 10.2307/1442678

[B7] Balon E. K. Stewart D. J. (1983). Fish assemblages in a river with unusual gradient (Luongo, Africa - Zaire system), reflections on river zonation, and description of another new species. Environ. Biol. Fish. 9, 225–252. 10.1007/BF00692373

[B8] Böhlke J. E. Weitzman S. H. Menezes N. A. (1978). Estado atual da sistemática dos peixes de água doce da América do Sul. Acta Amazon. 8, 657–677.

[B9] Casatti L. Castro R. M. C. (1998). A fish community of the São Francisco River headwaters riffles, southeastern Brazil. Ichthyol. Explor. Freshw. 9, 229–242.

[B10] Castello L. Macedo M. N. (2016). Large-scale degradation of Amazonian freshwater ecosystems. Glob. Change Biol. 22, 990–1007. 10.1111/gcb.13173 26700407

[B11] Castello L. McGrath D. G. Hess L. L. Coe M. T. Lefebvre P. A. Petry P. (2013). The vulnerability of Amazon freshwater ecosystems. Conserv. Lett. 6, 217–229. 10.1111/conl.12008

[B12] Ceballos G. Ehrlich P. R. Barnosky A. D. García A. Pringle R. M. Palmer T. M. (2015). Accelerated modern human-induced species losses: entering the sixth mass extinction. Sci. Adv. 1:e1400253. 10.1126/sciadv.1400253 26601195 PMC4640606

[B13] Cella-Ribeiro A. da Costa Doria C. R. Dutka-Gianelli J. Alves H. Torrente-Vilara G. (2017). Temporal fish community responses to two cascade run-of-river dams in the Madeira River, Amazon basin. Ecohydrology 10, 1–11. 10.1002/eco.1889

[B14] Clausen R. York R. (2008). Global biodiversity decline of marine and freshwater fish: a cross-national analysis of economic, demographic, and ecological influences. Soc. Sci. Res. 37, 1310–1320. 10.1016/j.ssresearch.2007.10.002

[B15] Collen B. Whitton F. Dyer E. E. Baillie J. E. M. Cumberlidge N. Darwall W. R. T.. (2014). Global patterns of freshwater species diversity, threat and endemism. Glob. Ecol. Biogeogr. 23, 40–51. 10.1111/geb.12096 26430385 PMC4579866

[B16] Collins R. A. Ribeiro E. D. Sousa L. M. Hrbek T. (2018). Hydroelectric dams on the Xingu and Tapajós rivers imperil a diverse and unique fauna of suckermouth catfishes. Aquat. Conserv.: Mar. Freshw. Ecosyst.

[B17] Corander J. Marttinen P. Sirén J. Tang J. (2008). Enhanced Bayesian modelling in BAPS software for learning genetic structures of populations. BMC Bioinformatics 9:539. 10.1186/1471-2105-9-539 19087322 PMC2629778

[B18] Darwall W. R. T. Holland R. A. Smith K. G. Allen D. Brooks E. G. E. Katarya V. (2011). Implications of bias in conservation research and investment for freshwater species. Conserv. Lett. 4, 474–482. 10.1111/j.1755-263X.2011.00202.x

[B19] de Queiroz K. (2007). Species concepts and species delimitation. Systemat. Biol. 56, 879–886. 10.1080/10635150701701083 18027281

[B20] Excoffier L. Lischer H. E. L. (2010). Arlequin suite ver 3.5: a new series of programs to perform population genetics analyses under Linux and Windows. Mol. Ecol. Resour. 10, 564–567. 10.1111/j.1755-0998.2010.02847.x 21565059

[B21] Excoffier L. Smouse P. E. Quattro J. M. (1992). Analysis of molecular variance inferred from metric distances among DNA haplotypes: application to human mitochondrial DNA restriction data. Genetics 131, 479–491. 1644282 10.1093/genetics/131.2.479 PMC1205020

[B22] Fearnside P. M. (2012). Belo Monte Dam: A Spearhead for Brazil's dam Building Attack on Amazonia?, GWF Discussion Paper 1210. Technical report, Global Water Forum, Canberra, ACT.

[B23] Fearnside P. M. (2015). Amazon dams and waterways: Brazil's Tapajós Basin plans. Ambio 44, 426–439. 10.1007/s13280-015-0642-z 25794814 PMC4510327

[B24] Fearnside P. M. (2016). Brazilian politics threaten environmental policies. Science 353, 746–748. 10.1126/science.aag0254 27540150

[B25] Finer M. Jenkins C. N. (2012). Proliferation of hydroelectric dams in the Andean Amazon and implications for Andes-Amazon connectivity. PLoS ONE 7:e35126. 10.1371/journal.pone.0035126 22529979 PMC3329437

[B26] Fitzgerald D. B. Sabaj Perez M. H. de Sousa L. M. Gonçalves A. P. Rapp Py-Daniel L. Lujan N. K. (2018). Diversity and community structure of rapids-dwelling fishes of the Xingu River: implications for conservation amid large-scale hydroelectric development. Biol. Conserv. 222, 104–112. 10.1016/j.biocon.2018.04.002

[B27] Flausino Junior N. Machado F. A. Zuanon J. Ferreira E. J. G. (2016). The fish fauna of sessile hydrophytes stands (*Mourera* spp.: Podostemaceae) in the Dardanelos Waterfalls, Rio Aripuanã, Brazil. Aqua Int. J. Ichthyol. 22, 133–144.

[B28] Frederico R. G. Olden J. D. Zuanon J. (2016). Climate change sensitivity of threatened, and largely unprotected, Amazonian fishes. Aquat. Conserv. Mar. Freshw. Ecosyst. 26, 91–102. 10.1002/aqc.2658

[B29] Frederico R. G. Zuanon J. De Marco P. (2018). Amazon protected areas and its ability to protect stream-dwelling fish fauna. Biol. Conserv. 219, 12–19. 10.1016/j.biocon.2017.12.032

[B30] Hawkins C. P. Kershner J. L. Bisson P. A. Bryant M. D. Decker L. M. Gregory S. V. (1993). A hierarchical approach to classifying stream habitat features. Fisheries 18, 3–12. 10.1577/1548-8446(1993)018 < 0003:AHATCS>2.0.CO;2

[B31] Hora S. L. (1930). Ecology, bionomics and evolution of the torrential fauna, with special reference to the organs of attachment. Philos. Trans. R. Soc. Lond. Ser. B 218, 171–282. 10.1098/rstb.1930.0005

[B32] International Energy Agency (2013a). World Energy Outlook – 2013. Paris: International Energy Agency.

[B33] International Energy Agency (2013b). World Energy Resources – 2013 Survey. Paris: International Energy Agency.

[B34] Isbrücker I. J. H. Nijssen H. (1991). *Hypancistrus zebra*, a new genus and species of uniquely pigmented ancistrinae loricariid fish from the Rio Xingu, Brazil (Pisces: Siluriformes: Loricariidae). Ichthyol. Explor. Freshw. 1, 345–350.

[B35] Jégu M. (1992). *Ossubtus xinguensis*, nouveaux genre et espèce du Rio Xingu, Amazonie, Brésil (Teleostei: Serrasalmidae). Ichthyol. Explor. Freshw. 3, 235–252.

[B36] Jégu M. Santos G. M. d. (1988). Une nouvelle espèce du genre *Mylesinus* (Pisces, Serrasalmidae), *Mylesinus paucisquamatus*, décrite du bassin du rio Tocantins (Amazonie, Brésil). Cybium 12, 331–341.

[B37] Jégu M. L. A. M. F. (2004). Taxinomie des Serrasalminae Phytophages et Phylogenie des Serrasalminae (Teleostei: Characiformes: Characidae). Ph.D. thesis, Paris.

[B38] Kearse M. Moir R. Wilson A. Stones-Havas S. Cheung M. Sturrock S.. (2012). Geneious Basic: an integrated and extendable desktop software platform for the organization and analysis of sequence data. Bioinformatics 28, 1647–1649. 10.1093/bioinformatics/bts199 22543367 PMC3371832

[B39] Kullander S. O. (1988). *Teleocichla*, a new genus of South American rheophilic cichlid fishes with six new species (Teleostei: Cichlidae). Copeia 1988, 196–230. 10.2307/1445938

[B40] Kullander S. O. (1991). *Crenicichla phaiospilus* and *C. percina*, two new species of pike cichlids (Teleostei: Cichlidae) from the rio Xingu, Brazil. Ichthyol. Explor. Freshw. 1, 351–360.

[B41] Latrubesse E. M. Arima E. Y. Dunne T. Park E. Baker V. R. D'Horta F. M.. (2017). Damming the rivers of the Amazon basin. Nature 546, 363–369. 10.1038/nature22333 28617466

[B42] Lees A. C. Peres C. A. Fearnside P. M. Schneider M. Zuanon J. A. S. (2016). Hydropower and the future of Amazonian biodiversity. Biodivers. Conserv. 25, 451–466.

[B43] Lévêque C. Oberdorff T. Paugy D. Stiassny M. L. J. Tedesco P. A. (2007). Global diversity of fish (Pisces) in freshwater. Hydrobiologia 595, 545–567. 10.1007/s10750-007-9034-0

[B44] Lowe-McConnell R. H. (1987). Ecological Studies of Tropical Fish Communities. Cambridge: Cambridge University Press.

[B45] Lujan N. K. Cramer C. A. Covain R. Fisch-Muller S. López-Fernández H. (2017). Multilocus molecular phylogeny of the ornamental wood-eating catfishes (Siluriformes, Loricariidae, *Panaqolus* and *Panaque*) reveals undescribed diversity and parapatric clades. Mol. Phylogenet. Evol. 109, 321–336. 10.1016/j.ympev.2016.12.040 28065866

[B46] Lujan N. K. Winemiller K. O. Armbruster J. W. (2012). Trophic diversity in the evolution and community assembly of loricariid catfishes. BMC Evol. Biol. 12:124. 10.1186/1471-2148-12-124 22835218 PMC3497581

[B47] Lundberg J. G. Kottelat M. Smith G. R. Stiassny M. L. J. Gill A. C. (2000). So many fishes, so little time: an overview of recent ichthyological discovery in continental waters. Ann. Missouri Bot. Garden 87, 26–62. 10.2307/2666207

[B48] Machado V. N. Collins R. A. Ota R. P. Andrade M. C. Farias I. P. Hrbek T. (2018). One thousand DNA barcodes of piranhas and pacus reveal geographic structure and unrecognised diversity in the Amazon. Sci. Rep. 8:8387. 10.1038/s41598-018-26550-x 29849152 PMC5976771

[B49] Markert J. A. Schelly R. C. Stiassny M. L. J. (2010). Genetic isolation and morphological divergence mediated by high-energy rapids in two cichlid genera from the lower Congo rapids. BMC Evol. Biol. 10:149. 10.1186/1471-2148-10-149 20482864 PMC2886069

[B50] Menezes N. A. (1996). Methods for assessing freshwater fish diversity, in Biodiversity in Brazil - A First Approach, eds de Bicudo C. E. Menezes N. A. (Brasília: CNPq), 289–296.

[B51] Meyer A. (1993). Evolution of mitochondrial DNA in fishes, in Molecular Biology Frontiers: Biochemistry and Molecular Biology of Fishes. Vol. 2 eds Hochachka P. W. Mommsen T. P. (Amsterdam: Elsevier), 1–38.

[B52] Pelicice F. M. Agostinho A. A. (2008). Fish-passage facilities as ecological traps in large Neotropical rivers. Conserv. Biol. 22, 180–188. 10.1111/j.1523-1739.2007.00849.x 18254863

[B53] Pelicice F. M. Azevedo-Santos V. M. Vitule J. R. S. Orsi M. L. Lima Junior D. P. Magalhães A. L. B. (2017). Neotropical freshwater fishes imperilled by unsustainable policies. Fish Fish. 18, 1119–1133. 10.1111/faf.12228

[B54] Platt A. R. Woodhall R. W. George A. L. Jr. (2007). Improved DNA sequencing quality and efficiency using an optimized fast cycle sequencing protocol. Biotechniques 43, 58–62. 10.2144/000112499 17695253

[B55] R Development Core Team (2011). R: A Language and Environment for Statistical Computing. Vienna: R Foundation for Statistical Computing.

[B56] Reis R. E. Kullander S. O. Ferraris Jr C. J. (2003). Check List of the Freshwater Fishes of South and Central America. Porto Alegre: EDIPUCRS.

[B57] Roberts T. R. Stewart D. J. (1976). An ecological and systematic survey of fishes in the rapids of the lower Zaïre or Congo River. Bull. Museum Comp. Zool. Harvard Univ. 147, 239–317.

[B58] Roxo F. F. Lujan N. K. Tagliacollo V. A. Waltz B. T. Gabriel S. Silva C.. (2017). Shift from slow- to fast-water habitats accelerates lineage and phenotype evolution in a clade of Neotropical suckermouth catfishes (Loricariidae: hypoptopomatinae). PLoS ONE 12:e0178240. 10.1371/journal.pone.0178240 28591189 PMC5462362

[B59] Salzburger W. Ewing G. B. Von Haeseler A. (2011). The performance of phylogenetic algorithms in estimating haplotype genealogies with migration. Mol. Ecol. 20, 1952–1963. 10.1111/j.1365-294X.2011.05066.x 21457168

[B60] Salzburger W. Van Bocxlaer B. Cohen A. S. (2014). Ecology and evolution of the African Great Lakes and their faunas. Ann. Rev. Ecol. Evol. Systemat. 45, 519–545. 10.1146/annurev-ecolsys-120213-091804

[B61] Sambrook J. Russell D. (2001). Molecular Cloning: A Laboratory Manual, 3rd Edn. Cold Springs Harbor, NY: Cold Springs Harbor Laboratory Press.

[B62] Santos G. M. D. Jégu M. (1987). Novas ocorrências de *Gnathodolus bidens, Synaptolaemus cingulatus* e descrição de duas espécies novas de *Sartor* (Characiformes, Anostomidae). Amazoniana 10, 181–196.

[B63] Santos G. M. D. Pinto S. S. Jégu M. (1997). Alimentação do pacu-cana, *Mylesinus paraschomburgkii* (Teleostei, Serrasalmidae) em rios da Amazônia brasileira. Rev. Brasil. Biol. 57, 311–315.

[B64] Simpson G. G. (1961). Principles of Animal Taxonomy. New York, NY: Columbia University Press. 10.1126/science.133.3464.1589 17781120

[B65] Soulé M. E. Wilcox B. (1980). Conservation Biology: An Evolutionary-Ecological Perspective. Sunderland, MA: Sinauer Associates, Inc.

[B66] Stamatakis A. (2014). RAxML version 8: a tool for phylogenetic analysis and post-analysis of large phylogenies. Bioinformatics 30, 1312–1313. 10.1093/bioinformatics/btu033 24451623 PMC3998144

[B67] Tajima F. (1989). Statistical method for testing the neutral mutation hypothesis by DNA polymorphism. Genetics 123, 585–595. 2513255 10.1093/genetics/123.3.585 PMC1203831

[B68] Thompson J. D. Higgins D. G. Gibson T. J. (1994). Clustal W: improving the sensitivity of progressive multiple sequence alignment through sequence weighting, position-specific gap and weight matrix choice. Nucleic Acids Res. 22, 4673–4680. 10.1093/nar/22.22.4673 7984417 PMC308517

[B69] Vitorino Júnior O. B. Agostinho C. S. Pelicice F. M. (2016). Ecology of *Mylesinus paucisquamatus* Jégu & Santos, 1988, an endangered fish species from the rio Tocantins basin. Neotrop. Ichthyol. 14:e150124 10.1590/1982-0224-20150124

[B70] Vörösmarty C. J. McIntyre P. B. Gessner M. O. Dudgeon D. Prusevich A. Green P.. (2010). Global threats to human water security and river biodiversity. Nature 467, 555–561. 10.1038/nature09440 20882010

[B71] Werle E. Schneider C. Renner M. Völker M. Fiehn W. (1994). Convenient single-step, one tube purification of PCR products for direct sequencing. Nucleic Acids Res. 22, 4354–4355. 10.1093/nar/22.20.4354 7937169 PMC331970

[B72] Willis S. C. (2017). One species or four? Yes!…and, no. Or, arbitrary assignment of lineages to species obscures the diversification processes of Neotropical fishes. PLoS ONE 12:e0172349. 10.1371/journal.pone.0172349 28235096 PMC5325279

[B73] Winemiller K. O. McIntyre P. B. Castello L. Fluet-Chouinard E. Giarrizzo T. Nam S.. (2016). Balancing hydropower and biodiversity in the Amazon, Congo, and Mekong. Science 351, 128–129. 10.1126/science.aac7082 26744397

[B74] Zuanon J. A. S. (1999). História Natural da Ictiofauna de Corredeiras do rio Xingú, na região de Altamira, Pará. Ph.D. thesis, Campinas.

